# Cognition around the world

**DOI:** 10.3389/fpsyg.2014.01149

**Published:** 2014-10-10

**Authors:** Barbara Götsch

**Affiliations:** Institute for Social Anthropology, Austrian Academy of SciencesVienna, Austria

**Keywords:** cognition, psychology, socio-cultural anthropology, universality, cultural variability

*Die Welt des Denkens: Kognitive Einheit, kulturelle Vielfalt*, may be translated as “The world of thinking: cognitive unity, cultural diversity.” It represents an impressive and much needed effort in German at bringing together insights from psychology and socio-cultural anthropology about how people around the globe perceive and order the world and how they feel and reason about the world surrounding them. The book is extraordinary in both scope and depth, it is lucidly written and, thanks to its thoughtful structure, it is accessible for a broad audience.

Eight areas that mental activity is directed at are addressed in particular: (1) color perception (2) the classification of plants and animals (3) logical reasoning (4) counting and calculating (5) spatial reasoning (6) reasoning about time (6) navigating the sea (7) social cognition and perspective taking (8) emotions. The different areas are treated in distinct chapters, preceded by an introduction outlining the scholarly study of cognition and culture and giving an overview of the book, and followed by a comprehensive conclusion and three different indices, including one on “countries, languages, and cultures.”

Very much in the style of a textbook, the individual chapters all follow a similar structure. They provide information on a cognitive phenomenon, first detailing what is known about it from a Western psychological or natural science perspective, and then putting this in relation with studies that may roughly be grouped as appertaining to the field of cognitive anthropology. The latter focus on how people in remote places of the world approach the same issue. As one might expect the respective results look quite different. The authors then engage with this difference and draw conclusions about the way culture and cognition interact. This is supplemented with a one page info box detailing general ethnographic knowledge about the specific society that appears to do this or that differently. In addition, each paragraph is complemented with keywords in the margins, pointing to the main information presented. The chapters are concluded with two service sections: one offering tasks or thought experiments that the reader may perform, the other providing suggestions for further reading.

We learn, for example, that the Tarahumara in Mexico use only one term to denote blue and green (siyó), and that the Inca, rather than using a script, used a highly differentiated notation system (quipu) of strings and knots to calculate and archive numerical knowledge concerning the community. We also learn that Australian Aborigines have a nearly perfect sense of direction in vast places such as the desert, where non-Aborigines would hardly find their way. This seems to result from a preference for an absolute frame of reference according to north, east, south and west rather than a relative one (as in “left,” “right”), which is also reflected in language and gesture. In addition, they orient themselves according to specificities in the landscape, which are laden with cultural memories. Samoa, then, is given as an example of a place where people refrain from overt speculation about the thoughts and feelings of others, which in turn has consequences for the way causality and accountability are explained.

The authors come to the conclusion that no clear-cut picture can be drawn as to the exact interrelation of culture and cognition. This is to do with difficulties in the methodological comparability of studies, the lack of studies addressing similar questions about people in different parts of the world, and generally the sheer scope of the problem, i.e., what aspect of cognition in relation to what aspect of culture is being addressed.

Some circumstances are described as consequential, however. Cultural preferences and environmental requirements seem to create differences in the ways people categorize, quantify and explain the world, and in how they orient themselves in the world. Also, schooling and literacy seem to have a strong influence on the ways in which the world is classified and causality is explained; furthermore, many recent and more refined studies support the old argument that language influences thought and perception (as in spatial reasoning). Overall, the authors argue that many differences in reasoning activity point to differences in quantity rather than quality, i.e., that the cognitive abilities are indeed universal but used in various styles, intensities and at different points in a person's development. This means for example that the Aboriginal sense of direction could be learned by members of another society, and that the Samoans could learn to impute the mental states of others at an earlier age were they encouraged to do so.

The authors, thus, come to the conclusion that human cognition is indeed strongly universal, allowing for some cultural variability. To my mind, from an anthropological perspective that is, the book somewhat ambivalently stresses the dichotomy of universality vs. cultural variability. This dichotomy shines forth on the title page, when at the same time the introductory chapter makes clear that things are not as universal as they appear to be. Also, the authors refer to the debate about the computer-inspired metaphor of content and process, and state that this distinction is artificial when it comes to the relationship between culture and the mind/body. By contrast, some recent anthropological thinking about the dynamic historical and biosocial character of human life rather tries to do away with this dual perspective and tries to highlight the developmental—and simultaneously cultural—plasticity of the mind and the body (Astuti and Bloch, 2010; Ingold and Palsson, 2013).

Along this line, the role of participation in social events, distributed cognition and embodiment could have been elaborated some more. Two areas that are highly interesting but are missing altogether as a topic in their own right are intercultural differences in approaches to causal reasoning and problem solving. They are touched upon in discussions about logical reasoning, navigation, perspective taking, and emotion, but are waiting to be explored further. It is therefore not surprising that the authors initiated a research program on the cultural constitution of causal cognition and co-edited a special issue of *Frontiers in Psychology* on this topic.

The book is structured pedagogically and discusses a vast amount of literature from the growing interdisciplinary field of cognition and culture. It seems particularly designed for students of psychology in their specialization phase but it will also appeal to students and teachers of socio-cultural anthropology, linguistics, and human biology, as well as to readers from other areas of the cognitive sciences, or from fields such as human geography and human ecology.

To conclude, the book aims at illustrating the intricate relationship between cognition and culture, and opens up an opportunity for dialogue between the disciplines of psychology and anthropology. It is highly informative and very well-structured. In its effort to try to give answers, it neglects to ask questions and theorize, however; which—at least for anthropologists—would make this book an even better read.

## Conflict of interest statement

The author declares that the research was conducted in the absence of any commercial or financial relationships that could be construed as a potential conflict of interest.
